# Ecological assembly of mucosal and fecal microbiomes in ulcerative colitis across genes, functions, and species

**DOI:** 10.3389/fmicb.2026.1851847

**Published:** 2026-08-14

**Authors:** Wendy Li, Yali Yuan, Xuewen Li, Jieshang Zhou

**Affiliations:** 1 College of Biological Sciences and Technology, Taiyuan Normal University, Taiyuan, China; 2 People’s Hospital of Dingxi City, Dingxi, China

**Keywords:** metagenomic function (MF), metagenomic gene (MG), mucosal microbiome, neutral community model, ulcerative colitis (UC)

## Abstract

**Introduction:**

Inflammatory bowel disease (IBD) is associated with gut microbial dysbiosis, yet the ecological processes underlying community assembly remain unclear.

**Methods:**

Here, we applied Sloan’s neutral community model to examine the relative contributions of stochastic and deterministic processes across multiple ecological layers, including metagenomic genes (MGs), molecular functions (MFs), and microbial species (archaea, bacteria, fungi, and viruses), in fecal and mucosal microbiota from healthy controls and ulcerative colitis (UC) patients.

**Results:**

Across all layers, most MGs, MFs, and species conformed to neutral expectations, indicating a dominant role of stochastic processes in community assembly. However, UC was consistently associated with an increased proportion of neutral MFs and species, accompanied by a reduction in non-neutral components, suggesting weakened selective constraints under disease conditions. Ecological niche further modulated these patterns. Fecal communities harbored significantly higher proportions of non-neutral MFs and species compared to mucosal communities, indicating stronger deterministic processes in feces. Analysis of selection-state transitions revealed extensive changes in selection status in UC, particularly in mucosal environments, where both functional and taxonomic profiles showed marked transition. These changes were primarily driven by the gain and loss of selection rather than directional switching, indicating that UC alters the strength and presence of selection rather than reversing its direction.

**Discussion:**

Together, these findings provide a multi-layered ecological framework for understanding UC-associated dysbiosis, highlighting increased neutrality and selection rewiring as key features of microbial community assembly under disease conditions.

## Introduction

Inflammatory Bowel Disease (IBD), encompassing Crohn’s disease (CD) and ulcerative colitis (UC), is a chronic, relapsing–remitting disorder of the gastrointestinal tract whose rising global incidence underscores the critical interplay between environmental factors and host biology. The disease etiology is multifactorial, arising from dysregulated immune responses in a genetically susceptible host, with the gut microbiome established as a central contributor to pathogenesis (Zhang et al., 2025). A hallmark of IBD is the disruption of gut microbial community structure, characterized by shifts in the abundance, diversity, and functionality of resident microorganisms. These alterations extend beyond bacteria to include archaea, fungi, and viruses, suggesting that IBD-associated dysbiosis is a cross-kingdom ecological phenomenon rather than a purely bacterial disorder. Understanding the ecological forces that govern the assembly of these diverse microbial communities in health and disease is therefore essential for elucidating the mechanisms that drive IBD pathogenesis (Valdés-Mas et al., 2025).

Research on the gut microbiome in IBD has traditionally focused on bacteria, where dysbiosis is characterized by reduced diversity, depletion of beneficial taxa such as *Faecalibacterium prausnitzii*, and expansion of pathobionts including adherent-invasive *Escherichia coli* (AIEC) (Sokol et al., 2008, 2020; Darfeuille-Michaud et al., 2004). Functionally, impaired production of short-chain fatty acids (SCFAs), particularly butyrate, and disrupted tryptophan metabolism contribute to epithelial dysfunction and mucosal inflammation (Lavelle and Sokol, 2020; Nikolaus et al., 2017). Beyond bacteria, archaeal taxa such as *Methanobrevibacter smithii* have been implicated in altered gut motility and inflammatory responses (Pennisi, 2017). Increasing evidence also highlights important roles for fungi and viruses in IBD pathogenesis. For example, *Debaryomyces hansenii* impairs mucosal healing in CD by activating a macrophage-mediated type I interferon-CCL5 axis, and *Malassezia restricta* exacerbates colitis through interaction with the host IBD-risk gene *CARD9* (Jain et al., 2021; Limon et al., 2019). Likewise, viruses, particularly bacteriophages, can modulate bacterial community structure and function (Houshyar et al., 2026); enrichment of *Caudovirales* phages (Zuo et al., 2019; Clooney et al., 2019) and phage-mediated suppression of bacterial anti-inflammatory pathways have been reported in IBD. Collectively, these findings indicate that IBD-associated dysbiosis is a cross-kingdom ecological phenomenon involving coordinated alterations in bacterial, archaeal, fungal, and viral communities.

Host genetics provide the susceptible background that shapes this ecosystem. Polymorphisms in genes like *CARD9* and *ATG16L1* disrupt beneficial host–microbe dialogues, impairing the production of protective metabolites (Lamas et al., 2016; Chu et al., 2016). The integration of multi-omics data (genomics, metabolomics, proteomics) has been powerful in defining these functional networks, revealing that dysbiosis in active IBD is a coordinated event affecting multiple metabolic pathways simultaneously (Lloyd-Price et al., 2019).

Despite these advances, a critical gap remains in understanding the *ecological assembly rules* that govern the microbiome’s structure and function in IBD. Community assembly is shaped by a balance of stochastic (neutral) processes—such as random birth, death, and dispersal—and deterministic (niche-based) processes, like host selection. Hubbell’s Unified Neutral Theory of Biodiversity provides a null model where community composition is driven primarily by stochasticity (Hubbell, 2001). Sloan et al. (2006, 2007) adapted this into a continuous “near-neutral” model for microbial communities, which defines taxa or genes as neutral, positively selected (above-neutral), or negatively selected (below-neutral). This model has been widely applied to study prokaryotic communities across diverse environments, including human, hot spring, lung, and animal gut microbiomes (Venkataraman et al., 2015; Li and Ma, 2020a, 2020b; Ma, 2021a, 2021b, 2024; Ma and Ellison, 2018). Its significance lies in providing a quantitative baseline to test the relative contributions of stochastic and deterministic processes in shaping microbial communities, even when specific environmental drivers are not directly observable. But remains underutilized in deciphering the assembly of the IBD microbiome across its full biological hierarchy.

Crucially, previous ecological analyses in IBD have largely focused on taxonomic profiles, lacking integration with the functional genetic potential (Iliev et al., 2025; Aldars-García et al., 2021). The assembly mechanisms governing metagenomic genes (MGs) and, more importantly, aggregated metagenomic functions (MFs) representing specific metabolic pathways in IBD are virtually unexplored. Furthermore, comparative analyses between fecal and mucosal-associated communities—which occupy distinct biogeographical niches—are needed to understand spatial ecological dynamics in health and disease. Fecal and mucosal microbiomes represent fundamentally different ecological habitats (Donaldson et al., 2016; Tropini et al., 2017). The intestinal lumen, from which fecal samples are derived, is a relatively open, nutrient-rich environment characterized by high microbial biomass, rapid turnover, and exposure to dietary substrates and oxygen gradients (Donaldson et al., 2016). In contrast, the mucosal surface represents the critical host–microbe interface, where microorganisms interact directly with the epithelial barrier, immune cells, and mucus layer. The mucosa is characterized by stronger host-mediated selection pressures, including antimicrobial peptide secretion, immunoglobulin A (IgA) binding, nutrient competition, and oxygen limitation, all of which impose deterministic filters on community assembly (Lavelle and Sokol, 2020). These distinct ecological conditions are expected to produce different balances of stochastic and deterministic assembly processes. Comparative analyses of fecal and mucosal microbiota therefore provide complementary insights into the spatial organization of the gut ecosystem and the mechanisms by which host–microbe interactions are altered in UC.

To address these gaps, this study applies Sloan’s neutral community model across multiple, integrated layers of shotgun metagenomic data from matched fecal and mucosal samples of healthy controls and IBD patients. We leverage this framework to systematically analyze the assembly of: (1) species-level abundances across all microbial kingdoms (bacteria, archaea, fungi, viruses); (2) individual metagenomic genes (MGs) representing the community’s genetic potential; and (3) metagenomic function (MFs) encoding key metabolic pathways implicated in UC, such as SCFA production and tryptophan metabolism. Our primary objective is to detect and quantify the shift in community assembly mechanisms—from neutral, dispersal-driven processes to deterministic, selection-driven processes—that characterizes the transformation from a healthy gut ecosystem to a dysbiotic state in UC. By identifying which specific taxa, genes, and pathways are subject to positive or negative selection in fecal versus mucosal niches, this study provides a unified ecological and functional perspective on the forces driving microbial dysbiosis in UC.

## Methods and datasets

### Sample collection

A total of 22 fecal samples and 42 intestinal mucosal samples were collected from 42 participants (21 healthy controls and 21 UC patients) aged 18–60 years in Kunming, China. UC diagnosis was established based on standard endoscopic, radiographic, and histological criteria, and all patients were treated with mesalazine. Mucosal samples were obtained from the rectum (~10 cm from the anus) using sterile biopsy forceps without prior bowel preparation and immediately frozen in liquid nitrogen, followed by storage at −80 °C until DNA extraction. Healthy controls had no history of gastrointestinal disease and had not received antibiotics prior to sampling. All procedures were approved by the Medical Ethics Board of the First People’s Hospital of Yunnan Province, and informed consent was obtained from all participants. Detailed information on the dataset is provided in Yu et al. (2026).

### DNA extraction, sequencing, and preprocessing

Qualified DNA extracted from each assayed sample was fragmented by nebulization. After end-repair and adapter ligation, short fragments were removed with Ampure beads. Library quantification was performed by Agilent 2100 Bioanalyzer and ABI StepOnePlus Real-Time PCR system. The qualified libraries were sequenced by using Illumina HiSeq™ platform. After removing adaptors, low quality and ambiguous bases from the raw reads, the remaining reads were aligned to human genome reference (GRCh38) by Bowtie2 with standard settings (Langmead and Salzberg, 2012). Finally, 119.23 Gb of high-quality pair-end reads for 64 samples were acquired with an average of 5.95 Gb per sample. Preprocessed reads were assembled *de novo* with SOAPdenovo2 (Luo et al., 2012) and Rabbit (You et al., 2013).

### Construction of gene, function and taxonomic profiles

MetaGeneMark (Lomsadze et al., 2010) v2.10 were employed to predict genes from the assembled contigs. Genes from different samples are combined and clustered using CD-Hit (sequence identity threshold 95% and alignment coverage threshold 90%) (Godzik and Li, 2006). A non-redundant catalog of 999,310 genes was constructed. The integrated gene catalogs were blasted against public databases, including NCBI non-redundant (Nr), Swiss-Prot, COG (Cluster of Orthologous Groups), KEGG (Kyoto Encyclopedia of Genes and Genomes), GO (Gene Ontology), eggNOG (Evolutionary Genealogy of Genes: Non-Supervised Orthologous Groups), and ARDB (Antibiotic Resistance Genes Database), to obtain functional annotations (with e-value < 1.0 × 10^−5^).

Sequencing reads were classified to the species-level using Kraken2 v2.1.2 with default parameters against the reference sequence databases (Wood et al., 2019). The reference sequence databases were built based on the complete bacterial, archaeal, viral and fungi genomes. After taxonomic classification, the species abundance was estimated using Bracken v2.6 (Lu and Salzberg, 2020; Lu et al., 2022).

### Sloan’s neutral theory model

The Sloan et al.’s (2006) neutral theory model was applied to infer the distribution of species abundance in the entire microbial community based on observations from limited samples. This model assumes that the local (target) community has reached saturation, with a constant total number of individuals (*N*). Within the target community, the death rate (*δ*) of individuals is assumed to be independent of species identity. Each dead individual is replaced either by immigration from a source community or by local reproduction within the target community. The probability that a dead individual is replaced by an immigrant from the source community is denoted as *m*, while the probability of replacement via local birth is (1 – *m*). Thus, community assembly and turnover are governed by the balance among migration, birth, and death processes. Let *x_i_* = *n*/*N* represent the occurrence frequency of species *i* across target community samples, where *n* is the number of samples in which species *i* is observed and *N* is the total number of samples. Let *p_i_* denote the relative abundance of species *i* in the source community. Under the Sloan’s model, *x* follows a beta distribution:


$$x_{i}\sim \text{Beta}[N_{T}mp_{i},N_{T}m(1-p)]$$

Based on this framework, the observed occurrence frequency of each species can be compared with the frequency predicted by the Sloan neutral model. By fitting the model using nonlinear regression, the migration parameter (*m*) can be estimated. Furthermore, by comparing predicted and observed frequencies, species can be classified into three categories: those consistent with neutral expectations, those with higher-than-predicted frequencies (indicative of positive selection), and those with lower-than-predicted frequencies (indicative of negative selection).

### Conceptual framework for MG- and MF-based neutral modeling

Sloan’s neutral model was originally developed to describe the assembly of taxonomic units through stochastic processes, including local reproduction, immigration, and loss (Sloan et al., 2006, 2007). In this study, we extended this framework to metagenomic genes (MGs) and metagenomic functions (MFs) by treating each gene or function as an operational metagenomic unit (OMU), analogous to operational taxonomic units in community ecology (Ma and Li, 2018; Ma, 2024). Under this framework, the abundance of an MG or MF within an individual sample represents its local occurrence, whereas all samples within a cohort collectively constitute the regional metacommunity (source pool). Unlike taxon-based models that track the dispersal of organisms, the OMU-based framework focuses on the distribution of genetic and functional traits across communities.

Under Sloan’s model, local communities are assumed to be saturated such that the loss (“death”) of a unit is immediately compensated by either local reproduction (probability 1 − *m*) or immigration from the regional metacommunity (probability *m*). The migration parameter (*m*) therefore reflects the probability that a gene or function detected in a local community originates from the regional metacommunity rather than local proliferation. For MGs, immigration primarily corresponds to the dispersal of microorganisms carrying a given gene among hosts, although horizontal gene transfer (HGT) may also contribute to the redistribution of specific genes. For MFs, immigration reflects the introduction of functional traits through microorganisms harboring genes associated with a given function. Conversely, local reproduction represents the proliferation of resident microorganisms and their associated genetic or functional repertoires within a community. The loss (“death”) process is interpreted as the depletion of genes or functions from a local community due to ecological drift, environmental filtering, or replacement by alternative taxa and traits. For MFs, this does not imply the complete disappearance of all constituent genes. Because most functions are encoded by multiple genes and may be shared across different taxa, functional persistence can be buffered by functional redundancy. Consequently, neutrality at the MF level reflects the aggregate stochastic dynamics of multiple constituent genes rather than the behavior of any single gene.

We acknowledge that genes and functions are not autonomous ecological entities in the same sense as species. Therefore, MG- and MF-based neutral models should be interpreted as describing the assembly dynamics of gene and functional repertoires embedded within microbial communities rather than the independent movement or selection of genes/functions themselves.

### Study design and comparison framework

All neutral model analyses were performed at three ecological levels: (i) metagenomic genes (MGs), capturing the genetic repertoire of the community; (ii) metagenomic functions (MFs), annotated against seven functional databases (COG, GO, KEGG, Nr, Swiss-Prot, ARDB, and eggNOG) and representing community-level functional potential; and (iii) microbial species, classified into archaea, bacteria, fungi, and viruses. Additionally, a whole-community analysis was conducted by integrating all microbial kingdoms into a single taxonomic dataset.

To systematically investigate microbial community assembly across ecological niches and disease states, samples were categorized into four groups based on sample type and health status: HM (mucosal samples from healthy cohorts), UM (mucosal samples from UC patients), HF (fecal samples from healthy cohorts), and UF (fecal samples from UC patients). Three primary comparative frameworks were performed: (1) healthy versus UC groups within the mucosal niche (HM vs. UM), (2) healthy versus UC groups within the fecal niche (HF vs. UF), and (3) mucosal versus fecal niches in both healthy and UC populations (HM vs. HF, UM vs. UF). For each ecological level, features (MGs, MFs, or species) were classified by the neutral model as below-neutral (negatively selected), neutral, or above-neutral (positively selected). The proportions of features in each selection category were compared between groups using Fisher’s exact tests, with *p*-values adjusted using the Benjamini–Hochberg false discovery rate (FDR) procedure.

To evaluate whether microbial species under different selection states differed in abundance, species were classified into three categories according to Sloan’s neutral model: below neutral (negatively selected), neutral, and above neutral (positively selected). Differences in species abundance among the three selection categories were first assessed using the Kruskal–Wallis rank-sum test. When significant differences were detected, pairwise comparisons were performed using Dunn’s *post hoc* test with Benjamini–Hochberg false discovery rate (FDR) correction for multiple testing. Statistical significance was defined as FDR-adjusted *p* < 0.05.

## Results

### Dataset overview and community characteristics

*De novo* assembly of the 64 metagenomes generated a non-redundant gene catalog comprising 999,310 metagenomic genes (MGs). Gene richness varied substantially among groups, with fecal communities harboring far larger MG repertoires than mucosal communities (HF: 837,461; UF: 702,766; HM: 177,958; UM: 67,369; Supplementary Table S1A), indicating markedly higher genomic diversity in the luminal niche. The MG catalog was functionally annotated against seven databases, including COG, GO, KEGG, Nr, Swiss-Prot, ARDB, and eggNOG, yielding thousands to hundreds of thousands of metagenomic functions (MFs) depending on the database (Supplementary Table S1B). Taxonomic profiling identified 3,740–8,046 microbial species across the four groups, including archaea (75–296 species), bacteria (3,079–6,654 species), fungi (60–66 species), and viruses (526–1,643 species) (Supplementary Table S1C).

Across all groups, bacteria represented the most species-rich kingdom, followed by viruses, whereas archaea and fungi contributed relatively small fractions of the microbiome. A pronounced niche effect was observed, with mucosal communities consistently exhibiting lower species richness than fecal communities across all microbial kingdoms (e.g., total species richness: HM = 5,996 vs. HF = 8,046; UM = 3,740 vs. UF = 7,870). Similar patterns were observed for MGs and MFs. Collectively, these results indicate that the intestinal mucosa harbors a substantially reduced but more selectively filtered microbial repertoire compared with the fecal environment, establishing a strong ecological contrast between the two niches for subsequent analyses.

### Neutrality, negatively selected, and positively selected microbial species

To assess the relative contributions of stochastic and deterministic processes in shaping microbial communities (archaea, bacteria, fungi, and viruses), Sloan’s neutral model was applied to the HF, UF, HM, and UM groups. Except for mucosal archaea, which failed to yield a reliable fit, all other models showed good performance (Table 1), indicating that neutrality broadly explains microbial species assembly.

**Table 1:** Fitting microbial of microbiome datasets to Sloan et al. (2006, 2007) neutral model.

Disease	Source community	Destination community	*N*	Immigration probability (*m*)	*R* ^2^	Total number of species	Percentage of negatively selected species (%)	Percentage of neutral species (%)	Percentage of positively selected species (%)
Archaea	HF	HF	5223.7	0.089	0.501	293	6.14	83.62	10.24
	UF	UF	1429.3	0.182	0.356	290	6.21	84.14	9.66
	HM	HM	25.7	0.458	−0.143	136	0.74	98.53	0.74
	UM	UM	6.9	0.404	−0.357	75	0.00	100.00	0.00
Bacteria	HF	HF	9209615.1	0.147	0.743	6,592	1.82	71.91	26.27
	UF	UF	7566463.5	0.113	0.634	6,551	3.08	79.13	17.78
	HM	HM	26297.5	0.364	0.680	5,227	2.35	92.48	5.17
	UM	UM	10216.2	0.157	0.812	3,079	2.27	96.30	1.43
Fungi	HF	HF	1369.2	0.153	0.540	66	1.52	39.39	59.09
	UF	UF	1048.0	0.150	0.481	66	6.06	53.03	40.91
	HM	HM	326.6	2.122	0.817	65	0.00	70.77	29.23
	UM	UM	357.6	1.680	0.868	60	0.00	75.00	25.00
Viruses	HF	HF	3536.4	0.189	0.394	1,095	5.11	89.22	5.66
	UF	UF	56218.2	0.006	0.420	963	6.96	88.47	4.57
	HM	HM	2282.0	1.277	0.854	568	0.18	83.98	15.85
	UM	UM	2484.0	1.233	0.852	526	1.52	83.08	15.40
Total	HF	HF	9219744.4	0.127	0.823	8,046	3.28	70.67	26.05
	UF	UF	7625159.0	0.105	0.726	7,870	4.47	78.54	16.99
	HM	HM	28931.7	0.553	0.761	5,996	1.62	93.18	5.20
	UM	UM	13064.7	0.384	0.855	3,740	2.78	95.21	2.01

**Figure 1:**
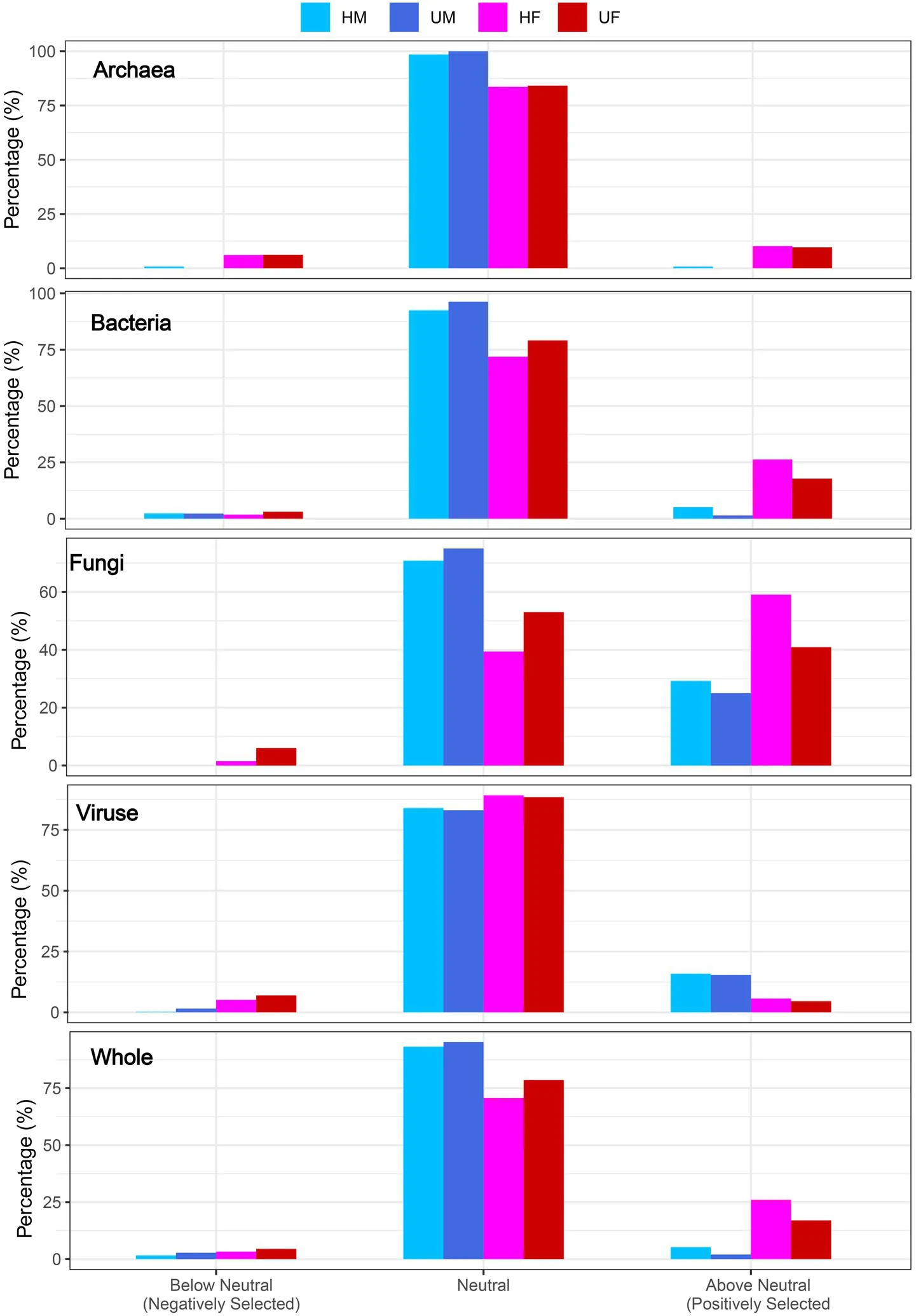
Percentages of below neutral (negatively selected), neutral, and above neutral (positively selected) microbial species across groups.

In fecal communities, most archaea, bacteria, and viruses were classified as neutral, with non-neutral species accounting for less than 30% (Figure 1). Notably, among non-neutral bacteria, the vast majority were under positive selection (HF: 93.52%; UF: 85.19%), suggesting strong adaptive processes. Fungi, however, displayed a distinct pattern. In HF, positively selected fungi dominated (59%), whereas in UF, neutral fungi became more prevalent (53.03%), accompanied by a reduction in positively selected species (40.91%). This indicates that deterministic processes—particularly positive selection—play a major role in shaping the fecal fungal community, but are weakened in UC. Disease-associated shifts were most evident in bacteria, where UC was associated with a significant increase in neutral species and a corresponding decrease in non-neutral species (HF vs. UF, non-neutral vs. neutral: Fisher’s exact test, *p* = 3.04 × 10^−22^, FDR < 0.001; Supplementary Table S2). More specifically, the proportion of negatively selected bacteria significantly increased (HF vs. UF, negatively selected vs. neutral: *p* = 1.17 × 10^−4^, FDR = 0.00054; Supplementary Table S2), while positively selected bacteria significantly decreased (HF vs. UF, positively selected vs. neutral: *p* = 2.16 × 10^−30^, FDR < 0.001; Supplementary Table S2). In fungi, the proportion of positively selected species also significantly decreased (HF vs. UF, positively selected vs. neutral: *p* = 0.046, FDR = 0.136; Supplementary Table S2).

Bacteria, fungi, and viruses in mucosal microbial communities are predominantly governed by neutral processes (Figure 1). Intriguingly, all non-neutral fungal species are under positive selection (HF: 100%; UF: 100%), and viruses show a similar trend (HF: 98.93%; UF: 91.02%), indicating that positive selection dominates the deterministic assembly processes of these two microbial groups. UC-associated alterations were mainly observed in bacteria and viruses. In bacteria, UC was characterized by a shift toward increased neutrality, with a reduction in non-neutral species. Specifically, the proportion of positively selected bacteria decreased sharply (HM vs. UM, positively selected vs. neutral: *p* = 1.92 × 10^−20^, FDR < 0.001; Supplementary Table S2). In contrast, in viruses, the primary change was an increase in negatively selected species in UC (HM vs. UM, positively selected vs. neutral: *p* = 0.015, FDR = 0.046; Supplementary Table S2).

Further analysis of the impact of ecological niches on community assembly reveals that the proportion of non-neutral bacteria and fungi is significantly higher in fecal samples than in mucosal samples (HM vs. HF, non-neutral vs. neutral: *p* < 0.001, FDR < 0.001; UM vs. UF, non-neutral vs. neutral: *p* < 0.001, FDR < 0.001), whereas viruses exhibit the opposite pattern, with non-neutral viruses being more prevalent in mucosal communities across both healthy (HM vs. HF, non-neutral vs. neutral: *p* = 0.0016, FDR = 0.0061) and UC (UM vs. UF, non-neutral vs. neutral: *p* < 0.0025, FDR = 0.0091; Supplementary Table S2) comparisons. Specifically, for bacteria and fungi, negatively selected species are more abundant in feces in 50% of comparisons, and positively selected species are significantly enriched in feces across all comparisons. Viruses display a unique niche-specific pattern: negatively selected viruses are consistently more abundant in feces (HF vs. HM, negatively selected vs. neutral: *p* < 0.001, FDR < 0.001; UF vs. UM, negatively selected vs. neutral: *p* < 0.001, FDR < 0.001), while positively selected viruses are significantly less abundant in feces than in mucosa (HF vs. HM, positively selected vs. neutral: *p* < 0.001, FDR < 0.001; UF vs. UM, positively selected vs. neutral: *p* < 0.001, FDR < 0.001; Supplementary Table S2), showing a distinct reversal in selection patterns.

**Figure 2:**
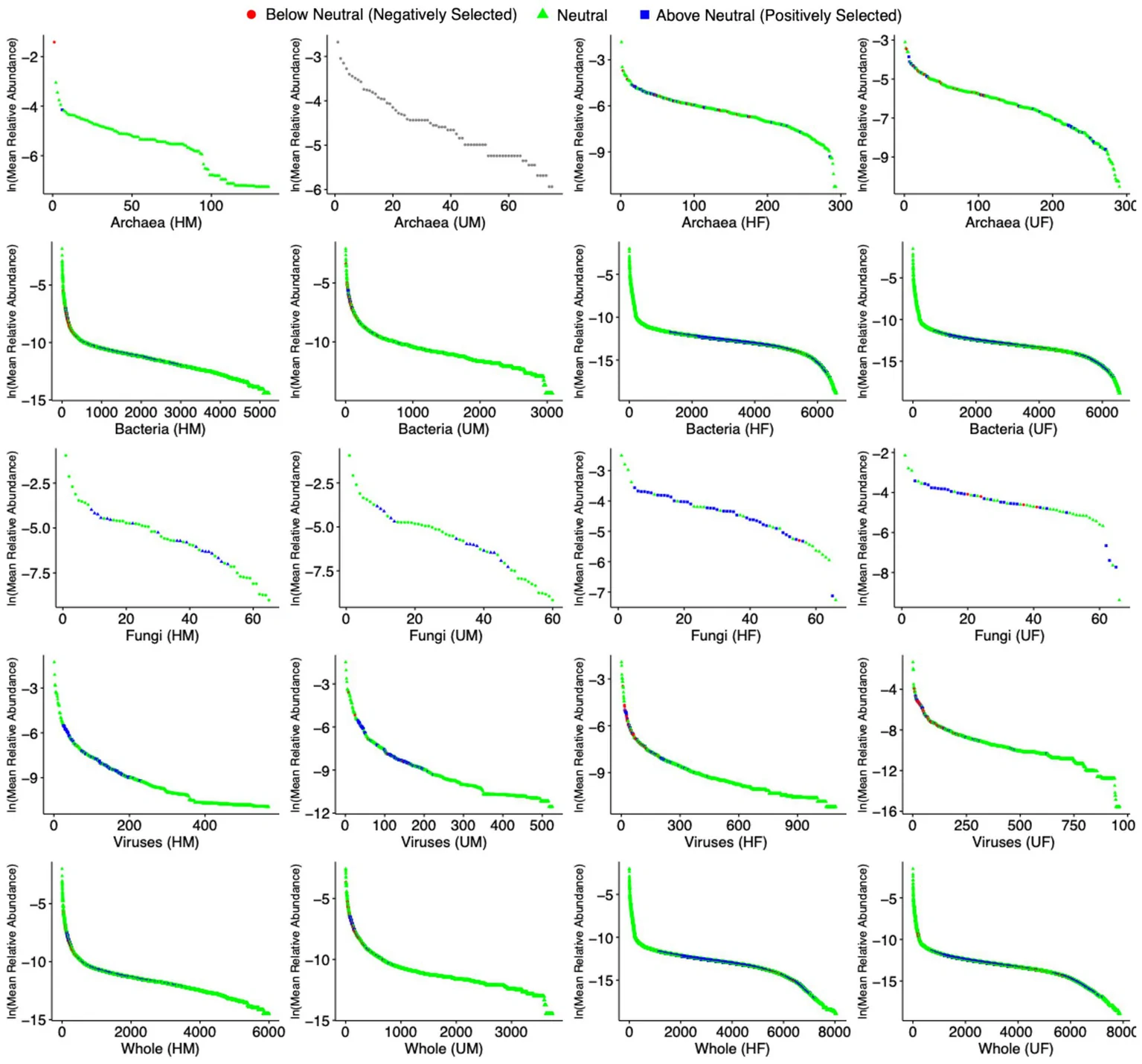
Relative abundance distribution of bacterial species across selection categories. Neutral species are shown as green triangles, below-neutral (negatively selected) species as royal blue circles, and above-neutral (positively selected) species as dark blue squares. The *y*-axis represents the logarithm of species relative abundance, and the *x*-axis indicates species rank based on relative abundance.

We further examined the abundance distribution of neutral, negatively selected, and positively selected species across niches and kingdoms (Figure 2). Distinct patterns emerged and were statistically evaluated using Kruskal-Wallis rank-sum tests followed by Dunn’s *post hoc* tests with Bonferroni correction (Supplementary Table S3, Supplementary Figure S1). In mucosal bacteria, mucosal viruses, fecal archaea, and fecal viruses, significant differences in abundance were detected among selection categories (Kruskal–Wallis tests, all FDR < 0.001). In these communities, non-neutral species generally exhibited higher abundances than neutral species. Specifically, both positively and negatively selected taxa were significantly more abundant than neutral taxa in most comparisons (Dunn’s tests, FDR < 0.001), although the comparison between positively selected and neutral archaea in UF and between negatively selected and neutral viruses in HM was not significant. These results indicate that deterministic processes preferentially act on dominant taxa in these communities. In fecal bacterial communities, the relationship between abundance and selection status differed between healthy and diseased states. In healthy feces (HF), neutral bacteria were significantly more abundant than both positively and negatively selected bacteria (Dunn’s tests, FDR < 0.001), indicating that dominant taxa were primarily governed by stochastic processes. In contrast, in UC feces (UF), positively selected bacteria were significantly more abundant than neutral bacteria (Dunn’s test, FDR < 0.001), suggesting that disease-associated environmental filtering preferentially promotes certain selected taxa. These findings indicate a shift from stochastic dominance in healthy fecal communities toward stronger deterministic regulation of abundant bacterial taxa in UC. In contrast, fungal communities exhibited much weaker abundance dependence. Significant differences among selection categories were detected only in UC feces (Kruskal–Wallis test, FDR = 0.039), where positively selected fungi were more abundant than neutral fungi (Dunn’s test, FDR = 0.034). No significant differences were observed in the remaining fungal communities. Collectively, these results indicate that deterministic selection is strongly associated with species abundance in archaea, bacteria, and particularly viruses, where non-neutral taxa are preferentially enriched among dominant community members. By contrast, fungal community assembly appears less dependent on species abundance, suggesting that the ecological consequences of selection differ substantially among microbial kingdoms.

### Neutrality, negatively selected, and positively selected MGs and MFs

To investigate the role of stochastic and deterministic factors in the assembly process of MGs and MFs (COG, GO, KEGG, Nr, Swissprot, ARDB, and eggNOG), Sloan’s neutral community model was fitted separately for the HF, UF, HM and UM groups.

**Figure 3:**
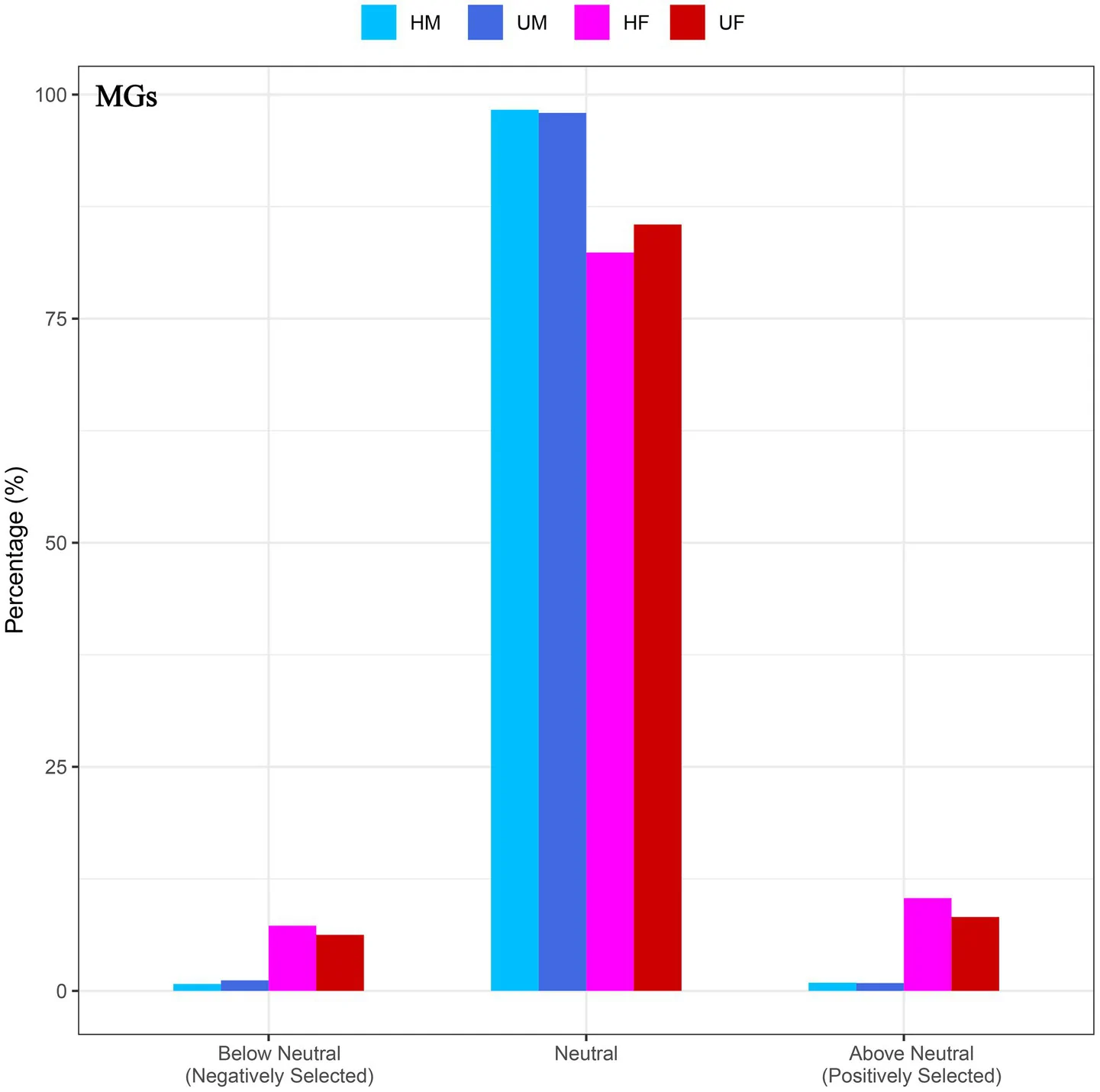
Percentages of below neutral (negatively selected), neutral, and above neutral (positively selected) metagenomic genes (MGs) across groups.

For MGs (Table 2), four neutral models were constructed. Acceptable model fits were observed only in mucosal samples, whereas the models for fecal samples performed poorly (*R*^2^ < 0), suggesting that the neutral model is insufficient to explain MG assembly in fecal communities. In mucosal microbiota, the vast majority of MGs were classified as neutral (HM: 98.31%; UM: 97.95%), indicating that stochastic processes dominate MG assembly (Figure 3). Only a small fraction (~1%) of MGs deviated from neutrality, reflecting the influence of deterministic processes, including both positive and negative selection. Notably, Fisher’s exact test revealed that the proportion of negatively selected MGs was significantly higher in UC mucosa compared to healthy controls (HM vs. UM, negatively selected vs. neutral: *p*-value = 4.03 × 10^−20^, FDR < 0.001; Supplementary Table S4), suggesting that UC mucosal microbiota accumulates more genes under negative selection constraints, potentially reflecting stronger environmental filtering or functional restriction.

**Table 2:** Fitting MGs of microbiome datasets to Sloan et al. (2006, 2007) neutral model.

Groups	Source community	Destination community	*N*	Immigration probability (*m*)	*R* ^2^	Total number of MGs	Percentage of negatively selected MGs (%)	Percentage of neutral MGs (%)	Percentage of positively selected MGs (%)
MGs	HF	HF	10525735.5	0.009	−0.080	837,461	7.27	82.38	10.35
	UF	UF	8151593.3	0.010	−0.025	702,766	6.25	85.50	8.24
	HM	HM	34689.2	0.478	0.484	177,958	0.77	98.31	0.92
	UM	UM	20715.6	0.446	0.643	67,369	1.17	97.95	0.87

**Figure 4:**
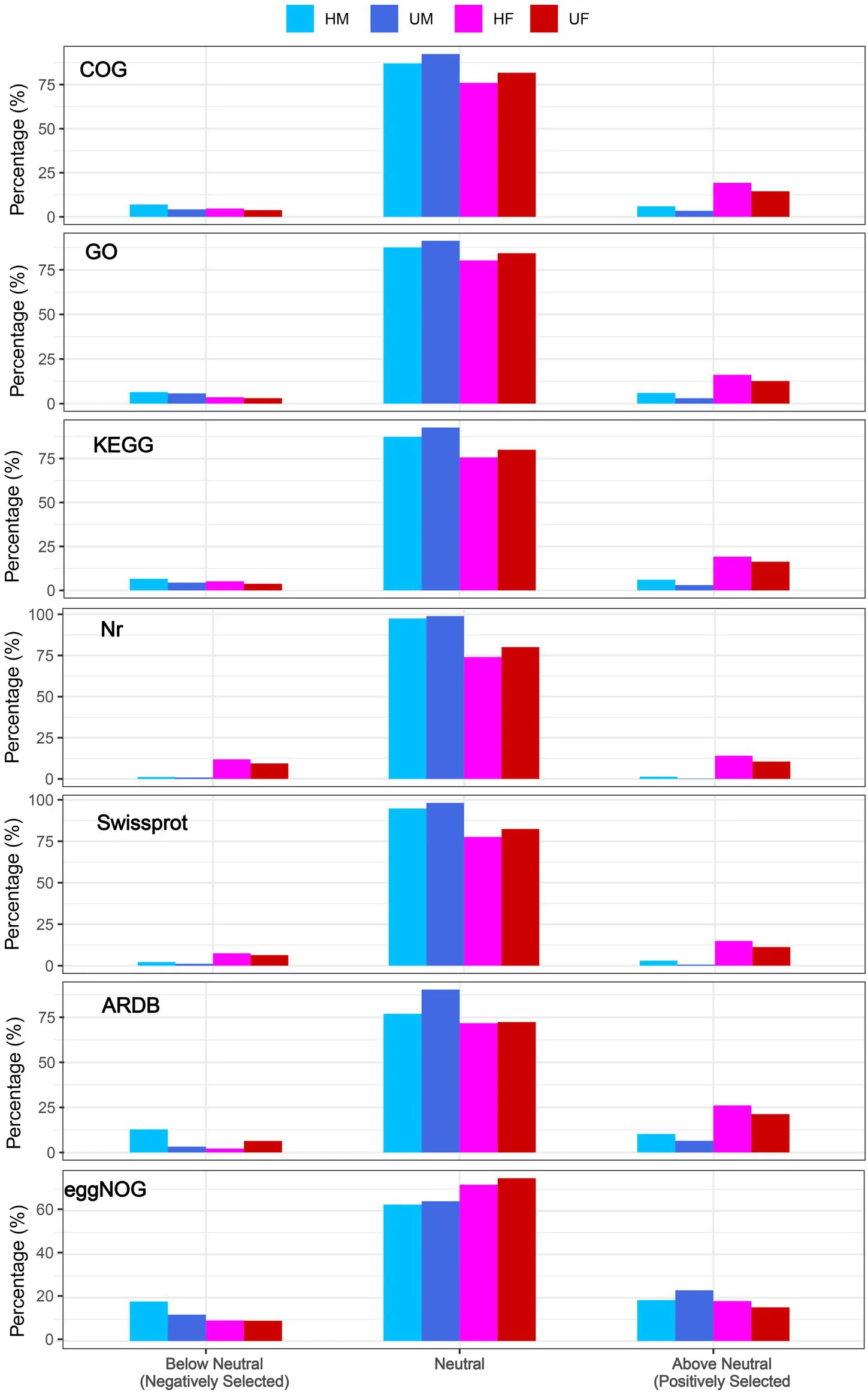
Percentages of below neutral (negatively selected), neutral, and above neutral (positively selected) metagenomic functions (MFs) across groups.

In contrast to MGs, all 28 neutral models for MFs showed good fits (Table 3), indicating that the neutral model broadly explains MF assembly across datasets. Consistently, most MFs were neutral (62.91–98.91%), suggesting a dominant role of stochastic processes (Figure 4). However, disease-associated differences were evident. Except for MFs from the ARDB database, the proportion of non-neutral MFs was significantly lower in UC compared to healthy controls (Fisher’s exact test, all FDR < 0.001; Supplementary Table S4), indicating that UC communities harbor a higher proportion of neutral MFs, i.e., functions less constrained by selection. More specifically: (*i*) Negatively selected MFs showed a consistent decrease in UC across both ecological niches. This reduction was statistically significant for all functional databases except GO, which showed a similar directional trend but did not remain significant after multiple-testing correction (HF vs. UF: *p* = 0.045, FDR = 0.112; HM vs. UM: *p* = 0.060, FDR = 0.143; Supplementary Table S4). (*ii*) Positively selected MFs also generally decreased in UC in both fecal and mucosal communities. However, an exception was observed in mucosal samples, where positively selected MFs derived from eggNOG were significantly enriched in UC (Fisher’s exact test, *p*-values < 0.001, FDR < 0.001; Supplementary Table S4).

**Table 3:** Fitting MFGCs of microbiome datasets to Sloan et al. (2006, 2007) neutral model.

Disease	Source community	Destination community	*N*	Immigration probability (*m*)	*R* ^2^	Total number of MFGCs	Percentage of negatively selected MFGCs (%)	Percentage of neutral MFGCs (%)	Percentage of positively selected MFGCs (%)
COG	HF	HF	2919607.8	0.024	0.809	3,260	4.72	75.98	19.29
	UF	UF	2343370.4	0.035	0.801	3,040	3.78	81.71	14.51
	HM	HM	7948.7	0.451	0.828	2,356	7.00	87.01	5.98
	UM	UM	2467.0	0.268	0.754	2075	4.24	92.34	3.42
GO	HF	HF	23658670.5	0.012	0.818	4,694	3.56	80.27	16.17
	UF	UF	19282888.2	0.021	0.818	4,628	3.05	84.29	12.66
	HM	HM	64455.3	0.378	0.866	3,749	6.46	87.54	6.00
	UM	UM	28008.4	0.222	0.827	3,366	5.73	91.27	3.00
KEGG	HF	HF	4807381.2	0.021	0.801	5,656	5.14	75.65	19.20
	UF	UF	3856362.7	0.029	0.787	5,189	3.74	79.96	16.30
	HM	HM	13536.8	0.425	0.832	3,915	6.59	87.36	6.05
	UM	UM	4504.8	0.250	0.756	3,487	4.39	92.60	3.01
Nr	HF	HF	9709226.8	0.015	0.233	250,306	11.89	74.02	14.09
	UF	UF	7680322.7	0.017	0.300	230,667	9.41	80.06	10.53
	HM	HM	28661.2	0.429	0.517	83,132	1.19	97.43	1.38
	UM	UM	15843.8	0.201	0.290	37,273	0.88	98.91	0.21
Swissprot	HF	HF	2699479.0	0.019	0.646	33,189	7.45	77.63	14.91
	UF	UF	2186647.8	0.025	0.685	31,342	6.38	82.39	11.23
	HM	HM	7689.3	0.516	0.742	12,638	2.20	94.74	3.06
	UM	UM	2845.4	0.320	0.547	8,158	1.19	98.14	0.67
ARDB	HF	HF	48326.2	0.020	0.799	46	2.17	71.74	26.09
	UF	UF	38671.8	0.018	0.798	47	6.38	72.34	21.28
	HM	HM	134.9	0.471	0.702	39	12.82	76.92	10.26
	UM	UM	43.5	0.428	0.632	31	3.23	90.32	6.45
eggNOG	HF	HF	5430729848.1	0.007	0.639	275,106	9.49	72.11	18.40
	UF	UF	4356774495.1	0.010	0.646	257,634	9.36	75.11	15.53
	HM	HM	16666662.9	0.040	0.465	193,059	18.23	62.91	18.86
	UM	UM	12279159.8	0.008	0.051	172,365	12.15	64.45	23.40

Additionally, the assembly of MF has niche-specific patterns, that deterministic processes play a more prominent role in fecal communities. In 78.6% (11/14) of comparisons, the proportion of non-neutral MFs was significantly higher in fecal samples than in mucosal samples (Supplementary Table S3). Similarly, positively selected MFs were more prevalent in feces in 71.42% (10/14) of comparisons. In contrast, negatively selected MFs showed inconsistent patterns: only 28.57% (4/14) of comparisons showed higher proportions in feces, while 35.71% (5/14) showed the opposite trend.

### Selection-state transitions of microbial species and metagenomic functions in UC

**Figure 5:**
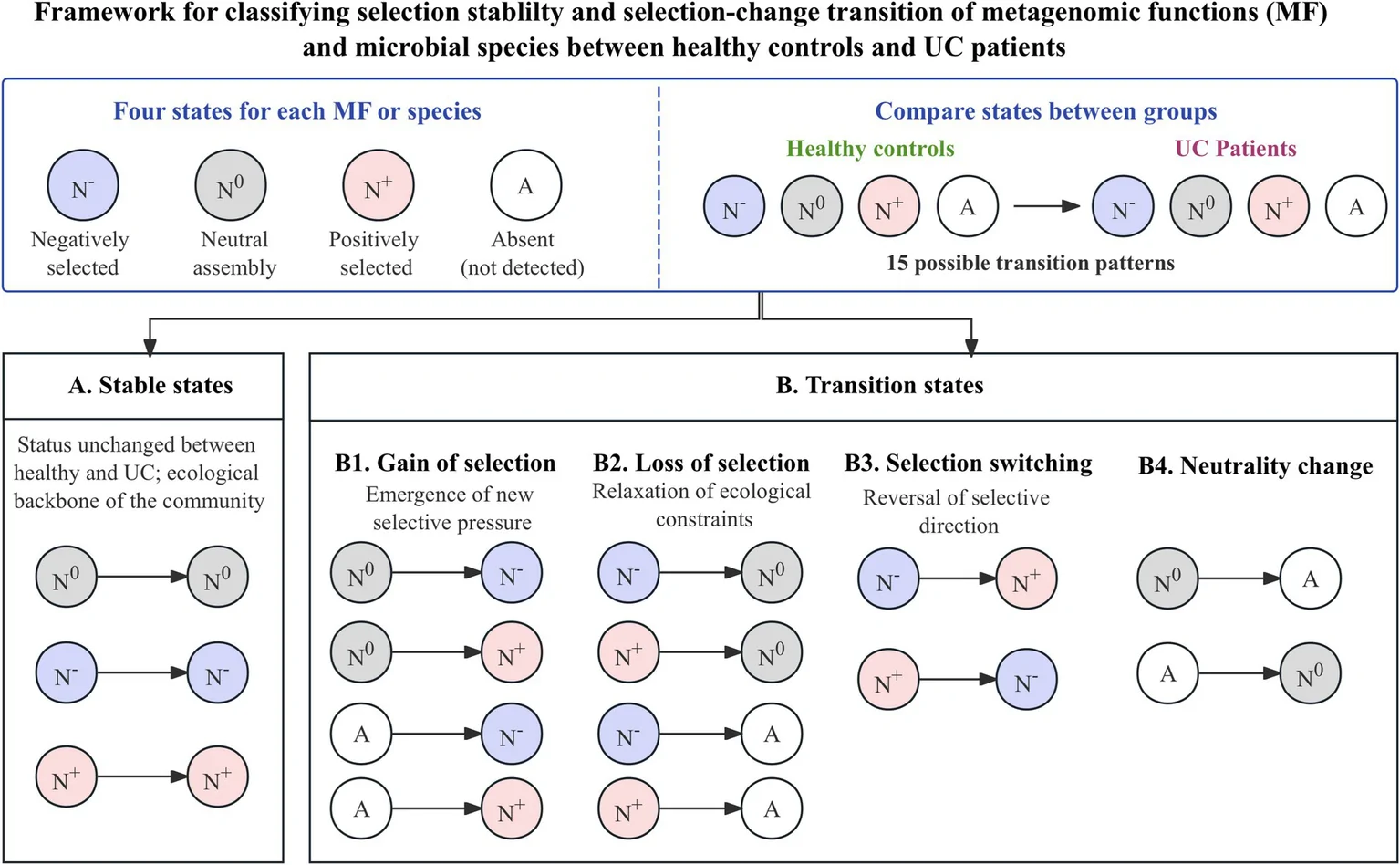
Classification framework for classifying disease-associated changes in selection status of functions and species in UC. Each MF or microbial species was assigned to one of four states in healthy controls and UC patients: Negatively selected, neutral, positively selected, or absent. Pairwise comparison between healthy and UC groups generated 15 possible transition patterns, which were classified into two major categories: Stable states and transition states. Stable states include neutral-to-neutral, negative-to-negative, and positive-to-positive transitions. Transition states comprise gain of selection, loss of selection, selection switching, and neutrality change.

To characterize how disease alters selection patterns, each MF and microbial species was assigned to one of four states within a given group: negatively selected, neutral, positively selected, or absent (not detected). We then compared the status of each feature between healthy controls and UC patients. A total of 15 selection-transition patterns are possible (Figure 5). These transitions were classified into two categories: stable states, in which a feature retained the same selection status between healthy and UC communities, and transition states, in which the selection status changed.

Stable states represent features whose status remains unchanged between healthy and UC communities and therefore may constitute a conserved ecological backbone. Three stable patterns were recognized: (*i*) neutral-to-neutral, (*ii*) negative-to-negative, and (*iii*) positive-to-positive. Transition states represent disease-associated changes in ecological assembly and can be grouped into four ecological processes. First, gain of selection (4 patterns) occurs when a neutral or absent feature becomes positively or negatively selected in UC, indicating the emergence of new selective pressures. Second, loss of selection (4 patterns) occurs when a selected feature becomes neutral or absent, suggesting relaxation of ecological constraints. Third, selection switching (2 patterns) refers to direct transitions between positive and negative selection, representing a reversal of selective direction. Finally, neutrality change (2 patterns) encompasses transitions between absent and neutral states, reflecting feature appearance or disappearance without evidence of deterministic selection. Detailed annotations and counts of all transition patterns are provided in Supplementary Tables S6–S7.

For microbial species, stable states accounted for 32.76–70.95% of species in fecal communities and 26.51–69.23% in mucosal communities (Supplementary Table S8). In archaea and bacteria, stable species were substantially less common in mucosa than in feces, suggesting stronger disease-associated perturbation of mucosal communities. Viral communities exhibited comparable levels of stability across niches, whereas fungi showed the opposite trend, with slightly higher stability in mucosa than in feces. For MFs, stable states accounted for 50.87–78.09% of functions in fecal communities and 38.19–63.64% in mucosal communities (Supplementary Table S9). Across all databases, the proportion of stable MFs was consistently lower in mucosa than in feces, indicating more extensive functional transition at the host–microbe interface during UC.

Importantly, across both MFs and microbial species, transitions involving gain or loss of selection were consistently more frequent than direct transitions between positive and negative selection (selection switching) across all functional databases and microbial kingdoms (Supplementary Tables S6–S7). Thus, UC-associated ecological reconfiguration appears to be driven primarily by the emergence or relaxation of selective pressures rather than by reversals in selective direction.

## Discussion

By applying Sloan’s neutral model across multiple biological levels, including microbial species, metagenomic genes (MGs), and metagenomic functions (MFs), this study provides an ecological perspective on gut microbiome assembly in ulcerative colitis (UC). Across taxonomic and functional layers, neutrality accounted for most community components, indicating that stochastic processes play a central role in shaping both healthy and diseased gut microbiomes. More importantly, UC was consistently associated with increased neutrality and reduced proportions of non-neutral species and functions, suggesting a relaxation of selective constraints during disease progression.

### Loss of selective pressure in the inflamed gut

Our key finding is the diminished role of deterministic selection in UC patients across virtually all analyzed components: microbial species, MGs, and MFs. Moreover, our transition analysis further showed that disease more frequently alters whether selection is present than whether a feature is favored or disfavored. In healthy individuals, microbial assembly is regulated by multiple host-derived selective forces, including immune surveillance, nutrient availability, epithelial integrity, and colonization resistance (Valdés-Mas et al., 2025). Chronic inflammation in UC disrupts these regulatory mechanisms and alters the ecological landscape of the gut, potentially weakening host-mediated selection and increasing the contribution of stochastic processes (Hubbell, 2001). This “ecological release” may permit the proliferation of pathobionts and the loss of keystone commensals not through competitive fitness, but simply through random population dynamics, explaining the increased inter-individual variability often observed in IBD microbiomes (Vandeputte et al., 2017). The observed increase in neutrality across both microbial species and MFs therefore suggests that UC is associated not only with compositional dysbiosis but also with a shift in the ecological processes governing community assembly.

### The mucosal niche: a locus of profound ecological change

A notable finding was the distinct ecological behavior of fecal and mucosal communities. Deterministic processes were generally stronger in fecal communities, which consistently contained higher proportions of non-neutral species and functions than mucosal communities. he mucosal drift towards neutrality in UC may be a key permissive factor allowing for the adhesion and invasion of pathobionts like AIEC, a process that requires a breakdown of the normally selective mucosal habitat. Furthermore, the altered virome and mycobiome in IBD, which our data show also become more neutrally assembled, likely contribute to this breakdown in niche control (Zuo et al., 2019; Jain et al., 2021; Wheeler et al., 2016). However, analyses of selection transitions revealed that UC-induced ecological reconfiguration was more pronounced at the mucosal interface. For both MFs and microbial species, the proportion of stable states was consistently lower in mucosal communities than in fecal communities, indicating that disease-associated changes in ecological assembly occur more extensively at the host–microbe interface. This pattern suggests that the mucosal niche, despite exhibiting weaker overall deterministic assembly, is more sensitive to disease-associated perturbation. Given that the intestinal mucosa is the primary site of immune surveillance and inflammatory activity in UC, alterations in host-derived selective pressures are likely to be manifested most strongly in this niche. Moreover, gain and loss of selection overwhelmingly exceeded direct switching between positive and negative selection, indicating that UC mainly modifies whether selection acts on a feature, rather than changing the direction of that selection.

### Functional implications of neutral assembly

The application of the neutral model to MFs provides unprecedented functional ecological insight (Ma and Li, 2018). The higher neutrality of metabolic pathways in UC patients suggests a decoupling of community function from host physiological needs. For instance, the reduced selection for SCFA-producing pathways aligns with the well-documented butyrate deficiency in IBD (Lavelle and Sokol, 2020). This is not simply a decrease in abundance of specific taxa like *F. prausnitzii* (Sokol et al., 2008), but an ecological shift where the functional genes themselves are no longer under host selection to maintain adequate production. Similarly, the altered assembly of virulence-associated genes underlines how loss of control can permit the random accumulation of detrimental functions. The dysregulation of critical immunomodulatory pathways, such as tryptophan metabolism into AhR ligands, may also stem from this broader ecological shift (Nikolaus et al., 2017; Lamas et al., 2016).

### Methodological advancements and study limitations

The extension of Sloan’s neutral model from taxonomic units to MGs and MFs is supported by the OMU framework, which treats genes and functions as ecological units analogous to OTUs (Ma and Li, 2018; Ma, 2021b, 2024). Under this framework, metagenome assemblages can be viewed as metacommunities in which genetic and functional traits are distributed among local gene pools through dispersal-like processes. Importantly, neutrality at the MG and MF levels does not imply that genes or functions behave as autonomous ecological entities. Rather, it reflects the stochastic assembly of community-level genetic and functional repertoires carried by microorganisms. Accordingly, immigration represents the introduction of genes or functional traits via dispersal of gene-carrying microorganisms (and potentially horizontal gene transfer), whereas local reproduction and loss correspond to the proliferation and depletion of the microbial populations harboring them. This interpretation is consistent with the notion that microbial ecological functions can become partially decoupled from taxonomic identity through gene gain, gene loss, and horizontal gene transfer, making function-based approaches a meaningful complement to taxon-centered analyses (Boon et al., 2014). Our results provide empirical support for this framework. The predominance of neutral MGs, MFs, and microbial species suggests that stochastic processes contribute substantially to gut microbiome assembly across multiple biological levels. Furthermore, the increased neutrality observed in both MFs and microbial species in UC is consistent with a relaxation of selective constraints under disease conditions.

Nevertheless, several limitations should be acknowledged. Functional categories aggregate signals from multiple taxa and may mask taxon-specific selection pressures, while functional redundancy and uniqueness are not explicitly distinguished. In addition, horizontal gene transfer may redistribute genes independently of organismal dispersal, weakening the direct correspondence between gene-level and species-level assembly processes. Therefore, MG- and MF-based neutral models should be interpreted as ecological approximations of community-level genetic and functional assembly rather than literal models of gene or function migration and selection.

Several datasets yielded poor fits to Sloan’s neutral model, most notably mucosal archaea and fecal MGs. We interpret these failures as arising from a combination of technical and biological factors. For mucosal archaea, the relatively low species richness (136 and 75 species in HM and UM, respectively) and low abundance may limit the occurrence-frequency distribution required for robust model fitting. In addition, archaeal populations in the human gut are typically rare, making abundance estimation more susceptible to sampling stochasticity. The near-complete neutrality observed in mucosal archaea (98.53–100% neutral species) further suggests that the limited number of non-neutral taxa may provide insufficient information for reliable parameter estimation. For fecal MGs, the extremely large number of genes (~10^6^) together with substantial inter-individual variation may generate occurrence-frequency distributions that deviate from the assumptions of Sloan’s model. Another possible explanation is the different level of biological organization represented by MGs. Unlike species or functional categories, individual genes are subject to gene-specific evolutionary processes, including gene gain and loss, horizontal gene transfer, and strain-level variation. These processes may produce distribution patterns that are not adequately captured by a neutral model originally developed for taxonomic assemblages. By contrast, MFs aggregate multiple genes with similar functions and are therefore buffered by functional redundancy, potentially explaining why MF-based models generally exhibited better fits than MG-based models. Importantly, poor model fit should not be interpreted solely as evidence for deterministic assembly. Rather, it indicates that the assumptions of the neutral model are insufficient to fully explain the observed distribution patterns. Additional ecological and evolutionary processes—including environmental filtering, host selection, functional redundancy, horizontal gene transfer, and stochastic drift operating at different biological scales—may all contribute to community assembly. Nevertheless, the failure of the neutral model remains biologically informative, as it identifies datasets in which neutrality alone cannot adequately account for community structure.

This study has several limitations. The cohort, while well-characterized, is from a single geographic region and consists solely of UC patients under mesalazine treatment, limiting generalizability to other populations, untreated patients, or Crohn’s disease. The cross-sectional design captures a snapshot of assembly states but cannot delineate causal sequences. While we identify a shift towards neutrality, the specific host factors (e.g., concentrations of specific cytokines, antimicrobial peptides) that correlate with this loss of selection remain to be defined. Furthermore, the model identifies the *outcome* of assembly processes (neutral vs. selected) but does not specify the exact deterministic factors (e.g., host genetics, dietary components) at play. Future studies integrating longitudinal sampling and host-derived multi-omics data may help elucidate the mechanisms linking host physiology to microbiome assembly processes (Lloyd-Price et al., 2019).

In conclusion, our findings indicate that UC is characterized by increased neutrality and reduced selective constraints across both taxonomic and functional dimensions of the gut microbiome. Although deterministic processes remain important, particularly in fecal communities, UC-associated ecological reconfiguration was more extensive in the mucosal niche, where both microbial species and metagenomic functions exhibited higher frequencies of selection-state transitions. These results suggest that UC-associated dysbiosis reflects not only altered community composition and functional potential, but also a broad reorganization of the ecological processes governing microbiome assembly, characterized by weakened selection and increased stochasticity. From a therapeutic perspective, our findings imply that successful microbiome-based interventions may require more than simply introducing beneficial microorganisms. Long-term restoration of a healthy microbiome may depend on re-establishing the host-derived selective forces that maintain ecological stability and support the persistence of beneficial taxa. Therapies that repair the mucosal barrier and alleviate intestinal inflammation may therefore contribute to microbiome recovery not only by modifying community composition, but also by restoring the selective environment that guides microbial assembly toward a stable, health-associated state.

## Data Availability

The original contributions presented in the study are included in the article/Supplementary material, further inquiries can be directed to the corresponding authors.
