# Supplementary material for: Ecological assembly of mucosal and fecal microbiomes in ulcerative colitis across genes, functions, and species
Source: Front Microbiol. 2026 Aug 14;17:1851847. doi: 10.3389/fmicb.2026.1851847 (PMC13522150; doi:10.3389/fmicb.2026.1851847)
Supplement: Supplementary file 1 [file Supplementary_file_2.DOCX]

**Supplementary Figure**

**Figure S1.** Comparison of species abundance among negatively selected (below neutral), neutral, and positively selected (above neutral) taxa in healthy and UC fecal and mucosal microbiomes.
